# FOXM1 recruits nuclear Aurora kinase A to participate in a positive feedback loop essential for the self-renewal of breast cancer stem cells

**DOI:** 10.1038/onc.2016.490

**Published:** 2017-01-23

**Authors:** N Yang, C Wang, Z Wang, S Zona, S-X Lin, X Wang, M Yan, F-M Zheng, S-S Li, B Xu, L Bella, J-S Yong, E W-F Lam, Q Liu

**Affiliations:** 1 State Key Laboratory of Oncology in South China, Cancer Center, Collaborative Innovation Center of Cancer Medicine, Sun Yat-Sen University, Guangzhou, China; 2 Institute of Cancer Stem Cell, Dalian Medical University, Dalian, China; 3 Department of Laboratory Medicine, Guangzhou First People’s Hospital, Guangzhou Medical College, Guangzhou, China; 4 Department of Surgery and Cancer, Imperial College London, Hammersmith Hospital, London, UK; 5 Department of Medical Oncology, The Eastern Hospital of the First Affiliated Hospital, Sun Yat-Sen University, Guangzhou, China

## Abstract

Substantial evidence suggests that breast cancer initiation, recurrence and drug resistance is supported by breast cancer stem cells (BCSCs). Recently, we reported a novel role of Aurora kinase A (AURKA) in BCSCs, as a transactivating co-factor in the induction of the c-Myc oncoprotein. However, the mode of action and transcriptional network of nuclear AURKA in BCSCs remain unknown. Here, we report that nuclear AURKA can be recruited by Forkhead box subclass M1 (FOXM1) as a co-factor to *trans*activate FOXM1 target genes in a kinase-independent manner. In addition, we show that AURKA and FOXM1 participate in a tightly coupled positive feedback loop to enhance BCSC phenotype. Indeed, kinase-dead AURKA can effectively *trans*activate the FOXM1 promoter through a Forkhead response element, whereas FOXM1 can activate AURKA expression at the transcriptional level in a similar manner. Consistently, breast cancer patient samples portrayed a strong and significant correlation between the expression levels of FOXM1 and AURKA. Moreover, both FOXM1 and AURKA were essential for maintaining the BCSC population. Finally, we demonstrated that the AURKA inhibitor AKI603 and FOXM1 inhibitor thiostrepton acted synergistically to inhibit cytoplasmic AURKA activity and disrupt the nuclear AURKA/FOXM1-positive feedback loop, respectively, resulting in a more effective inhibition of the tumorigenicity and self-renewal ability of BCSCs. Collectively, our study uncovers a previously unknown tightly coupled positive feedback signalling loop between AURKA and FOXM1, crucial for BCSC self-renewal. Remarkably, our data reveal a novel potential therapeutic strategy for targeting both the cytoplasmic and nuclear AURKA function to effectively eliminate BCSCs, so as to overcome both breast cancer and drug resistance.

## Introduction

Breast cancer is one of the principal causes of female mortality worldwide.^1^ Although surgery coupled with adjuvant chemotherapy largely improves survival rates, there is still a substantial portion of patients who are refractory to the present chemotherapeutic strategies. Substantial recent evidence suggests that breast cancer progression, recurrence and drug resistance are supported by the presence of breast cancer stem cells (BCSCs).^2^ However, the molecular mechanisms that govern self-renewal of BCSCs and drug resistance remain, to date, largely elusive. Understanding how BCSCs acquire the ability to self-renew and develop drug resistance will therefore greatly aid in the design of novel therapies targeted at eliminating these malignant cells.

Aurora kinases are highly conserved serine/threonine kinases.^3^ The canonical function of Aurora kinases is regulating centrosome duplication and separation by promoting mitotic spindle assembly.^4^ A large body of research indicates that Aurora kinase A (AURKA) overexpresses in a variety of tumours and endows them with uncontrolled mitosis.^4, 5^ Consistent with previous studies, along with others, we have uncovered that AURKA dysregulation is associated with tumour metastasis and chemotherapeutic resistance. In agreement, AURKA inhibition can suppress the proliferation of a diverse range of malignancies, including nasopharyngeal carcinoma, acute myeloid leukaemia and breast cancer.^6, 7, 8^ However, although these inhibitors exhibited a tolerable toxicity profile and promising initial clinical efficacy, most failed to surpass the initial trials.^9, 10, 11, 12^ Recently, we showed that these failures are caused by the non-kinase-dependent nuclear *trans*activating function that enhances cancer stem cell properties that drive drug resistance.^13^ Nevertheless, the complicated molecular regulatory network of nuclear AURKA still needs to be further elucidated.

Forkhead box (FOX) superfamily of proteins are responsible for the spatio-temporal regulation of a broad range of transcriptional programmes critical for normal homeostasis and development.^14^ The FOX subclass M1 (FOXM1) transcription factor is a key factor in cancer initiation, progression and drug resistance.^15^ We previously reported that FOXM1 is a key downstream effector of the PI3K-AKT, ATM/p53-E2F and p38-MAPK-MK2 signalling cascades involved in chemotherapeutic drug sensitivity and resistance in myriad of cancers,^16, 17, 18, 19, 20, 21, 22^ suggesting that FOXM1 could potentially be a useful molecular biomarker and a therapeutic target for the elimination of cancer stem cells.

Here we uncovered a previous unknown positive feedback loop between AURKA and FOXM1 crucial for the acquisition of self-renewal properties and drug resistance of BCSCs. We further demonstrated that the AURKA and FOXM1 inhibitors can function synergistically to inhibit AURKA activity and disrupt the positive feedback loop to more effectively limit the tumorigenicity of breast cancer cells. These new data propose a promising therapeutic strategy to effectively target AURKA function to eliminate BCSCs.

## Results

### AURKA promotes FOXM1 expression and increases the BCSC population

**Figure 1:**
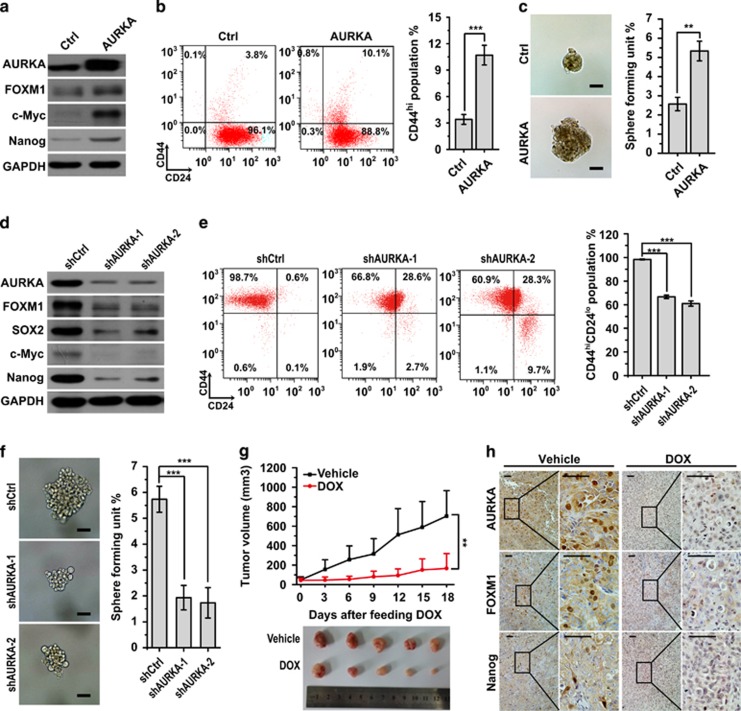
AURKA activates FOXM1 expression and increases BCSC sub-populations. (**a**) Western blot analysis with indicated antibodies in control (Ctrl) and AURKA overexpressing MCF-7 cells. (**b**, **c**) CD44^hi^ population and sphere (diameter >100 μm) formation in control (Ctrl) and AURKA overexpressed MCF-7 cells. Scale bar, 50 μm. (**d**) Western blot analysis with indicated antibodies in control (shCtrl) and AURKA (shAURKA) knockdown MDA-MB-231 cells. (**e**, **f**) CD44^hi^/CD24^lo^ population and sphere (diameter >100 μm) formation in control (shCtrl) and AURKA shRNA (shAURKA-1, shAURKA-2) knockdown MDA-MB-231 cells. (**g**) Nude mice bearing Dox-inducible AURKA knockdown MDA-MB-231 xenograft tumours were treated with Dox or vehicle (DMSO) every 3 days by intragastric administration for 18 days. The tumour volume was calculated. The cells were treated with 200 ng/ml Dox or vehicle (DMSO) for one week before transplanting to the nude mice. (**h**) IHC staining detected the expression of AURKA, FOXM1 and Nanog in tumour mass from **e**. Scale bar, 50μm. The above CD44^hi^/CD24^lo^ population and sphere formation assays were repeated at least three times independently and results presented as mean±s.d. **P<0.01; ***P<0.001; significance by Student's *t*-test (two tailed).

We previously showed that overexpression of AURKA was responsible for drug resistance and predicted an inferior prognosis in breast cancer.^13^ Here we revealed that AURKA overexpression significantly augmented the expression of FOXM1 and cancer stem cell markers c-Myc and Nanog in breast cancer cells (Figure 1a). We then examined whether AURKA participates in regulation of BCSCs. Overexpression of AURKA significantly increased the CD44^hi^ population and the mammosphere formation capacity in MCF-7 breast cancer cells (Figures 1b and c; Supplementary Figure S1A). Consistently, AURKA knockdown downregulated the expression of FOXM1 and that of the cancer stem cell markers c-Myc, SOX2 and Nanog in MDA-MB-231 cells (Figure 1d). Accordingly, knockdown of AURKA also reduced the CD44^hi^/CD24^lo^ sub-population and the mammosphere formation capacity in MDA-MB-231 breast cancer cells (Figures 1e and f; Supplementary Figure S1B).

Furthermore, we subcutaneously injected MDA-MB-231 cells harbouring stably integrated inducible AURKA knockdown short hairpin RNA (shRNA) constructs into nude mice and evaluated their effects on tumour growth. In comparison with vehicle (dimethylsulphoxide (DMSO))-treated control cells, which formed large tumours, doxycycline (Dox)-induced cells displayed substantially reduced tumour growth (Figure 1g). In addition, immunohistochemistry revealed a reduction in AURKA, FOXM1 and Nanog staining in Dox-treated tumours, compared with the DMSO-treated controls (Figure 1h). Finally, we performed limiting dilution assays in NOD/SCID mice to evaluate effect of AURKA on stemness *in vivo*. As shown in Table 1, AURKA knockdown reduced tumour incidence, indicating that AURKA has an essential function in tumour initiation.

### FOXM1 contributes to AURKA-mediated BCSC expansion

**Figure 2:**
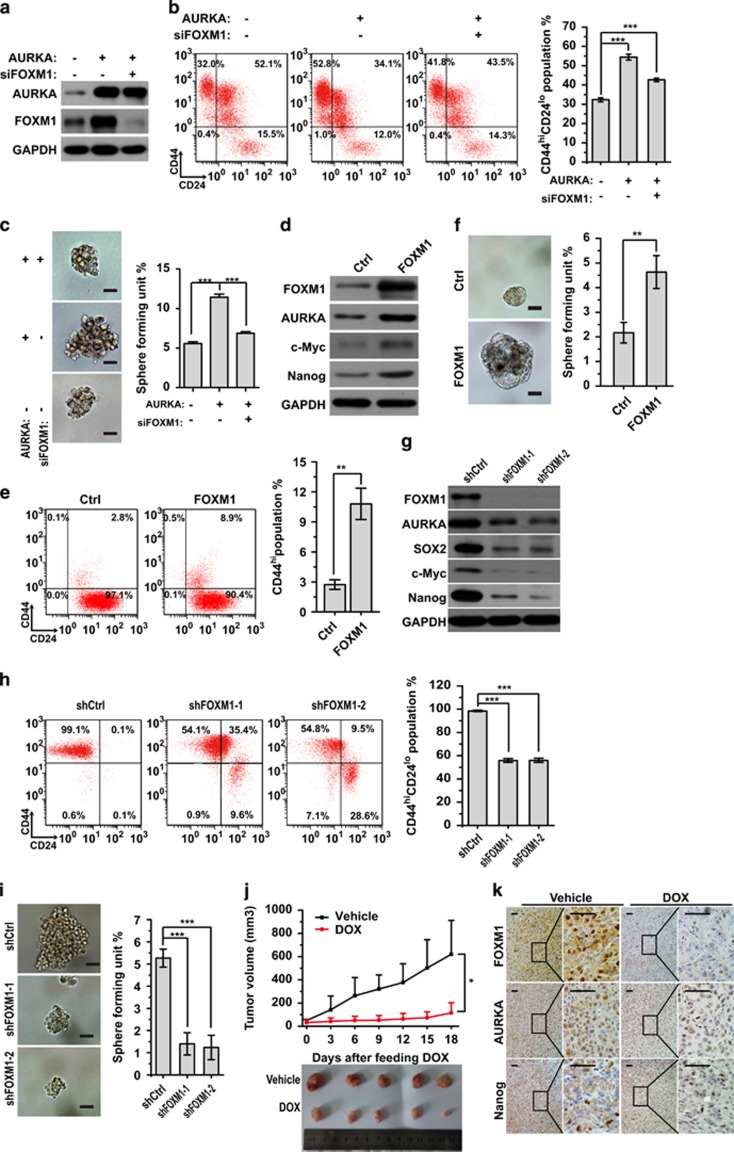
Overexpression of FOXM1 contributes to AURKA-mediated BCSC expansion. (**a**) Western blot analysis with indicated antibodies in control and AURKA overexpressing or FOXM1 knockdown SUM-149 cells. (**b**, **c**) CD44^hi^/CD24^lo^ population and sphere (diameter >100 μm) formation in control, AURKA overexpressed and AURKA overexpressed with FOXM1 knockdown SUM-149 cells. (**d**) Western blot analysis with indicated antibodies in control (Ctrl) and FOXM1 overexpressed MCF-7 cells. (**e**, **f**) The CD44^hi^ population and sphere (diameter >100 μm) formation in control (Ctrl) and FOXM1 overexpressed MCF-7 cells. (**g**) Western blot analyses with indicated antibodies in control (shCtrl) and FOXM1 (shFOXM1) knockdown MDA-MB-231 cells. (**h**, **i**) The CD44^hi^/CD24^lo^population and sphere (diameter >100 μm) formation in control (shCtrl) and FOXM1(shFOXM1) shRNA knockdown MDA-MB-231 cells. (**j**) Nude mice bearing inducible FOXM1 knockdown MDA-MB-231 xenograft tumours were treated with Dox, or vehicle (DMSO) every 3 days by intragastric administration for 18 days. The tumour volume was calculated. The cells were treated with 200 ng/ml Dox or vehicle (DMSO) for one week before transplanting to the nude mice. (**k**) IHC staining detected the expression of FOXM1, AURKA and Nanog in tumour mass from **j**. Scale bar, 50 μm. The above CD44^hi^/CD24^lo^ population and sphere formation assays were repeated at least three times independently and results presented as mean±s.d. **P<0.01; ***P<0.001 significance by Student's *t*-test (two tailed).

To investigate if FOXM1 is indeed a key downstream target of AURKA, we knocked down FOXM1 in Sum-149 cells that overexpressed AURKA and examined the stemness of BCSCs. The results showed that the positive effects of AURKA on the CD44^hi^/CD24^lo^ cell population and mammosphere formation were significantly impaired by FOXM1 depletion (Figures 2a–c). Next, we overexpressed FOXM1 and examined the stemness of BCSCs. The result showed that FOXM1 overexpression significantly increased the expression levels of AURKA as well as cancer stem cell markers c-Myc and Nanog, in MCF-7 cells (Figure 2d). Furthermore, overexpression of FOXM1 also significantly increased the CD44^hi^ fraction and the mammosphere formation capacity in MCF-7 cells (Figures 2e and f), whereas FOXM1 knockdown in MDA-MB-231 cells had the opposite effects (Supplementary Figure S2). Consistently, FOXM1 knockdown downregulated the expression levels of cancer stem cell markers SOX2, c-Myc and Nanog, and reduced the mammosphere formation capacity and the CD44^hi^/CD24^lo^ population in MDA-MB-231 cells (Figures 2g–i).

Furthermore, we subcutaneously injected MDA-MB-231 cells harbouring stably integrated inducible FOXM1 knockdown shRNA constructs into nude mice to evaluate their effects on tumour growth. In contrast to vehicle (DMSO)-treated control cells, which formed large tumours, Dox-induced cells gave rise to greatly reduced tumours (Figure 2j). Futhermore, immunohistochemical staining revealed decreased levels of FOXM1, AURKA and Nanog expression in Dox-induced tumours, compared with the control DMSO-treated cells (Figure 2k). Finally, we performed limiting dilution assays in NOD/SCID mice to evaluate effect of FOXM1 on stemness *in vivo*. As shown in Table 1, FOXM1 knockdown reduced tumour incidence, indicating that FOXM1 has a key role in tumour initiation. Collectively, these data implied that AURKA increases the proportion of BCSCs by positively regulating FOXM1.

### AURKA transcriptionally activates FOXM1 expression in kinase-independent manner

**Figure 3:**
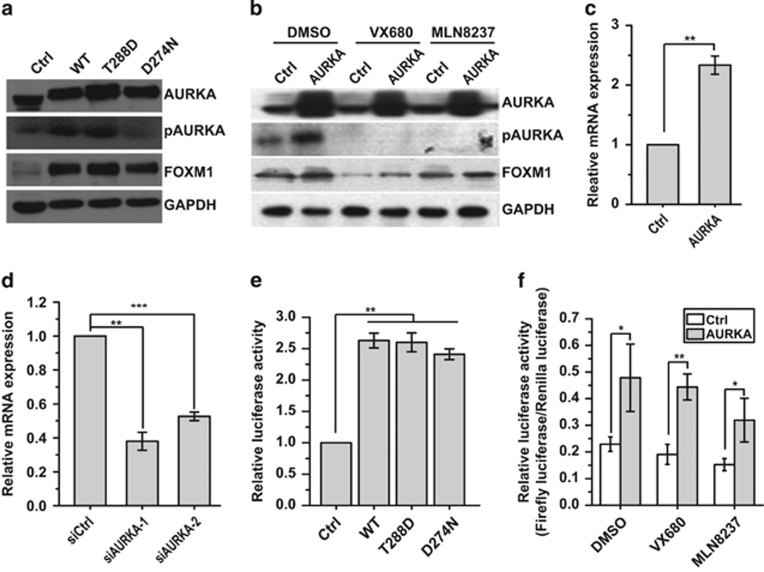
Nucleus AURKA transcriptionally activates FOXM1 expression in a kinase-independent manner. (**a**) Western blot analysis with indicated antibodies in control (Ctrl) and AURKA WT, kinase-activated form (T288D), or kinase-defective form (D274N) overexpressed MCF-7 cells. (**b**) Western blot analysis with indicated antibodies in AURKA overexpressed or control MDA-MB-231 cells treated with DMSO, VX680 and MLN8237. (**c**) Real-time PCR analysis of FOXM1 mRNA expression in AURKA overexpressing MCF-7 cells. (**d**) Real-time PCR analysis of FOXM1 mRNA expression in AURKA knocked down MCF-7 cells. (**e**) Luciferase reporter assay analysis of FOXM1 promoter activity in MCF-7 cells in the absence or presence of WT, kinase-mimicking, and kinase-defective AURKA overexpression. (**f**) Luciferase reporter assay analysis of FOXM1 promoter activity in AURKA overexpressing or control MDA-MB-231 cells treated with DMSO, VX680 and MLN8237. The above reverse transcriptase–quantitative PCR and promoter assays were repeated at least three times independently and results presented as mean±s.d. *P<0.05; **P<0.01; ***P<0.001 significance by Student's *t*-test (two tailed).

Given that both AURKA and FOXM1 are cell cycle regulatory genes involved in G2/M phase transition,^4, 23^ we therefore evaluated whether the canonical kinase activity of AURKA is essential for FOXM1 activation. Interestingly, both the AURKA-activated form (T288D) and the kinase-defective form (D274N) could promote FOXM1 expression (Figure 3a), suggesting that AURKA activates FOXM1 expression in a kinase-independent manner. To confirm this result, we inhibited AURKA kinase activity with the small molecule kinase inhibitors VX680 and MLN8237, respectively, and then studied the expression of FOXM1. As shown in Figure 3b, the expression of FOXM1 was significantly increased in AURKA overexpressed cells, although the active phosphorylated form of AURKA (P-AURKA) was hardly detectable. These results suggest that AURKA can activate FOXM1 expression independent of its kinase activity.

FOXM1 expression could be regulated at the transcriptional and post-transcriptional levels.^24^ We next examined the levels of FOXM1 mRNA in MCF-7 cells after AURKA overexpression or depletion. The results showed that the FOXM1 mRNA levels significantly increased when AURKA was overexpressed, and significantly decreased with AURKA knocked down (Figures 3c and d). In concordance, a similar phenomenon was observed in another breast cancer cell line MDA-MB-231 (Supplementary Figure S3). Furthermore, the luciferase reporter assay also showed that the AURKA wild-type (WT), kinase-activated form (T288D) and kinase-defective form (D274N) could all effectively enhance FOXM1 promoter activity (Figure 3e). Meanwhile, AURKA kinase inhibitors could not suppress the induction of FOXM1 expression by AURKA (Figure 3f). Interestingly, FOXM1 level was reduced by AURKA kinase inhibitors, indicating that AURKA kinase inhibitors have some effects on FOXM1 protein level. Collectively, these results suggest AURKA can transcriptionally activate FOXM1 expression independent of its kinase function.

### Nuclear AURKA binds directly to *FOXM1* promoter to *trans*activate its expression

**Figure 4:**
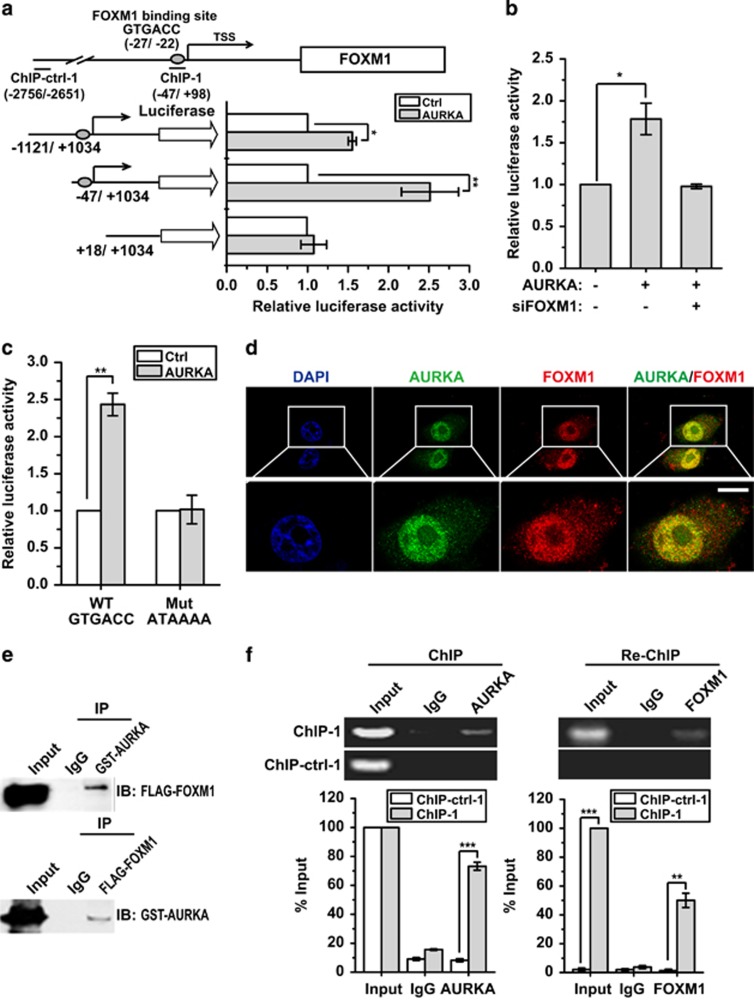
Nuclear AURKA binds directly to FOXM1 to *trans*activate FOXM1 expression. (**a**) Promoter truncation assay to locate the AURKA regulatory region in FOXM1 promoter. (**b**) Luciferase reporter assay analysis of FOXM1 promoter activity in MCF-7 cells with AURKA overexpression in the presence or absence of FOXM1 knockdown. (**c**) Luciferase reporter assay analysis of FOXM1 promoter activity with WT or mutated FOXM1-binding element (FHRE) in AURKA overexpressed or control MCF-7 cells. (**d**) Immunofluorescence assay to detect intracellular localisation of endogenous AURKA and FOXM1. Scale bar, 10 μm. (**e**) Co-IP assay to detect the interaction of AURKA and FOXM1. (**f**) ChIP Re-ChIP assay to analyse promoter co-occupation by FOXM1 and AURKA. The ChIP Re-ChIP assay was performed using chromatin prepared from MCF-7 cells. The chromatin was first precipitated with the anti-AURKA antibody or the control (IgG) and the precipitated chromatin analysed by reverse transcriptase (RT)–quantitative PCR (qPCR) using primers recognising the FHRE region (ChIP-1) and control primers (ChIP-ctrl-1) (4A) (left panel). The precipitated chromatin was then re-precipitated with the anti-FOXM1 antibody and the product analysed by RT–qPCR (right panel). The above promoter and ChIP RT–qPCR assays were repeated at least three times independently and results presented as mean±s.d. *P<0.05; **P<0.01; ***P<0.001; significance by Student's *t*-test (two tailed).

Next, we performed a promoter truncation assay to locate the AURKA responsive region in the *FOXM1* promoter. The result showed that the *trans*activation effect was lost when +1/+300 region was deleted (Figure 4a). Interestingly, transcription factor-binding sites analysis indicated that a consensus Forkhead-responsive element (FHRE) locates in this region, suggesting that AURKA may regulate FOXM1 expression through targeting *FOXM1* directly. Previous studies have reported that FOXM1 could bind its own promoter and *trans*activate its own expression. As AURKA cannot directly bind gene promoters, we hypothesised that AURKA activates *FOXM1* promoter through binding to FOXM1. To test this conjecture, we assessed the ability of AURKA to *trans*activate the *FOXM1* promoter with and without FOXM1 depletion. As revealed in Figure 4b, the ability of AURKA to enhance *FOXM1* promoter activity was lost in the absence of FOXM1, suggesting that AURKA induces FOXM1 expression via recruiting FOXM1. To corroborate this result, two types of point mutations were introduced into the FHRE of the *FOXM1* promoter to disrupt FOXM1 binding. The luciferase reporter assay showed that AURKA lost its *FOXM1* promoter activation effect while this motif was mutated (Figure 4c). Collectively, these results strongly suggested that AURKA can *trans*activate *FOXM1* expression through recruiting the FOXM1 protein.

To confirm further the interaction between AURKA and FOXM1, we performed confocal microscopy and co-immunoprecipitation (co-IP) assays. The results suggested that AURKA and FOXM1 colocalise in the nucleus and interact with each other (Figures 4d and e; Supplementary Figure S4). We further confirmed that AURKA and FOXM1 co-occupy the *FOXM1* gene promoter using ChIP Re-ChIP assay. The result suggested that AURKA complexes with FOXM1 on the FHRE of the *FOXM1* promoter (Figure 4f). Taken together, these results clearly show that FOXM1 recruits nuclear AURKA to directly *trans*activate *FOXM1* transcription.

### FOXM1 directly activates AURKA expression at the transcriptional level

**Figure 5:**
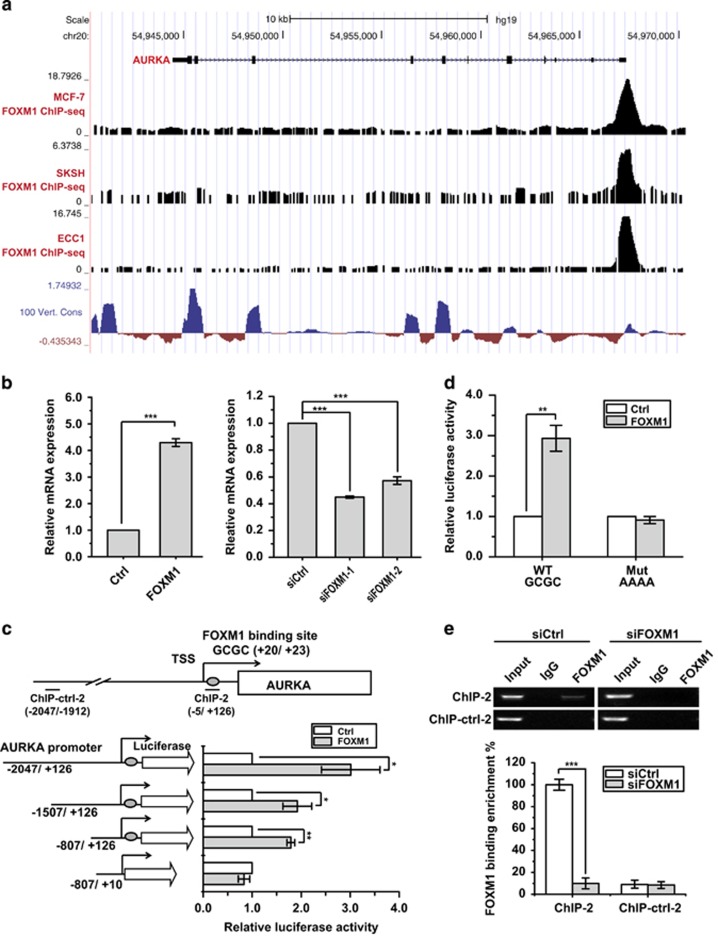
FOXM1 directly activates AURKA expression at the transcriptional level. (**a**) FOXM1 binding on AURKA promoter and gene body in MCF-7, SKSH and ECC1 cells. Data were obtained from ENCODE UCSC genome browser. 100 Vert. Cons tracks display multiple alignments of 100 vertebrate species and measurements of evolutionary conservation 49. (**b**) Real-time PCR analysis of AURKA mRNA expression in the presence or absence of FOXM1 overexpression and knockdown. (**c**) Promoter truncation assay to locate the FOXM1 regulatory region in the AURKA promoter. (**d**) Luciferase reporter assay analysis of AURKA promoter activity in MCF-7 cells with or without FOXM1 overexpression. (**e**) ChIP assay to analyse promoter occupation by FOXM1 on the AURKA promoter. The ChIP assay was performed using chromatin prepared from MCF-7 cells with or without FOXM1 depletion. The chromatin was first precipitated with the anti-FOXM1 antibody or the control (IgG) and the precipitated chromatin analysed by reverse transcriptase (RT)–quantitative PCR (qPCR) using primers recognising the FHRE region (ChIP-2) and control primers (ChIP-ctrl-2) (5C) (left panel). The precipitated chromatin was then analysed by RT–qPCR. The above promoter and ChIP RT–qPCR assays were repeated at least three times independently and results presented as mean±s.d. *P<0.05; **P<0.01; ***P<0.001; significance by Student's *t*-test (two tailed).

As shown in Figures 2d and g, AURKA showed a similar expression pattern as FOXM1 after we overexpressed or depleted FOXM1, suggesting that AURKA is regulated by FOXM1. We therefore analysed the recruitment of FOXM1 to the *AURKA* promoter using the published FOXM1 ChIP-seq data^25^ and ENCODE UCSC genome browser.^26, 27^ As shown in Figure 5a, FOXM1 binding was detected as a strong peak at the promoter region of *AURKA*, indicating that AURKA expression is directly regulated by FOXM1 at the promoter level. Next, we investigated the AURKA mRNA levels in FOXM1 overexpression and silencing cells, and observed that AURKA mRNA and protein levels significantly changed with FOXM1 expression (Figure 5b; Supplementary Figure S5). We then performed a promoter truncation analysis to locate the FOXM1 responsive region in the *AURKA* promoter. The data indicated that the *trans*activational effect of FOXM1 was diminished when the FHRE-containing region (−806/−28) was deleted (Figure 5c). To confirm this result, a mutation was introduced into the putative FHRE to disrupt FOXM1 binding. Indeed, FOXM1 lost its ability to *trans*activate the *AURKA* promoter when the FHRE was mutated (Figure 5d). To confirm whether AURKA promoter was a direct FOXM1 target, we performed ChIP analysis in MDA-MB-231 cells and observed strong FOXM1 binding at the promoter region containing the FHRE but not in the control promoter, an upstream site, which lacks a FHRE, or in the IgG-negative control (Figure 5e). These data suggested that FOXM1 binds directly to *AURKA* promoter to activate AURKA expression at the transcriptional level.

### AURKA and FOXM1 expression co-elevate in breast cancer

**Figure 6:**
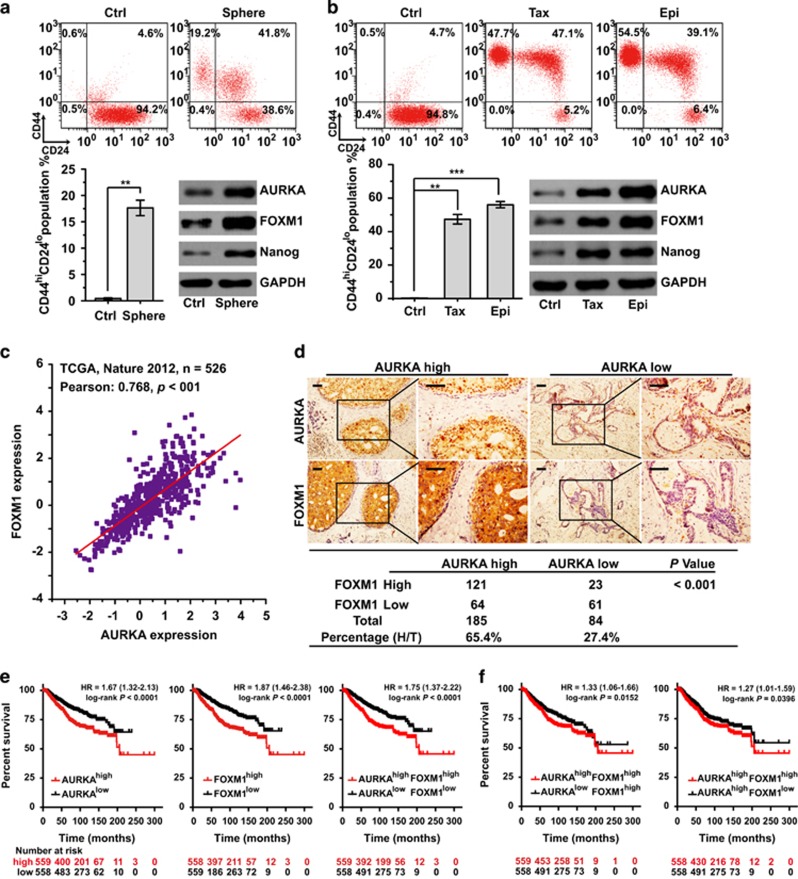
AURKA and FOXM1 co-expression in breast cancer. (**a**) Flow cytometry and western blot analysis of CD44^hi^/CD24^lo^ population as well as AURKA and FOXM1 expression in spheroid and control cells. (**b**) Flow cytometry and western blot analysis of CD44^hi^/CD24^lo^ population, as well as AURKA and FOXM1 expression in paclitaxel (TaxR) and epirubicin (EpiR) drug-resistant MCF-7 cells. (**c**) Expression of AURKA and FOXM1 in a TCGA batch consisting of 526 breast cancer patient samples. Pearson's correlation and linear regression analysis were employed. (**d**) Immunohistochemistry to determine protein expression levels of AURKA and FOXM1 in primary human breast cancer patient samples. Statistical comparisons were made using Chi-square test. (**e**, **f**) OS was examined by Kaplan–Meier analysis comparing the indicated subgroups. The above CD44^hi^/CD24^lo^population assays were repeated at least three times independently and results presented as mean±s.d. **P<0.01; ***P<0.001 significance by Student's *t*-test (two tailed).

To corroborate our findings, we examined the expression of AURKA and FOXM1 in BCSC-enriched spheroid cells, as well as in paclitaxel (TaxR) and epirubicin (EpiR)-resistant MCF-7 cells. The results indicated that both AURKA and FOXM1 were overexpressed in these BCSC-enriched spheroid and drug-resistant cells (Figures 6a and b).

To validate this finding further, we next studied the expressions of AURKA and FOXM1 in primary human breast cancer samples. We first analysed the correlations between AURKA and FOXM1 mRNA levels in a TCGA cohort, consisting of 526 breast cancer patient samples.^28^ Pearson's correlation indicated a strong and significantly positive correlation between the expression levels of AURKA and FOXM1 mRNA (Pearson coefficient=0.768, *P*<0.01, Figure 6c). Next, immunohistochemistry was used to determine protein levels of AURKA and FOXM1 in 269 primary human breast cancer patient samples from Sun Yat-Sen University Cancer Center. Clinical parameters and features of the patients and their samples are summarised in Supplementary Table S1. We detected high AURKA and FOXM1 expression in 121 of 185 (65.4%) specimens, indicating a significant positive correlation between AURKA and FOXM1 protein levels (Figure 6d). As the follow-up time of our breast cancer patient samples was not enough to analyse the clinical outcome, we explored AURKA/FOXM1 expression and prognosis in the Kaplan–Meier plotter database.^29^ As shown in Figure 6e, high expression of either AURKA, FOXM1 alone, or both significantly predicted poor overall survival (OS) in 1117 breast cancer patients. Interestingly, patients with high levels of AURKA and FOXM1 had significantly poorer OS outcomes when compared to those with high expression of AURKA or FOXM1 alone (Figure 6e).

Furthermore, we performed limiting dilution assays in NOD/SCID mice to compare the effect of AURKA and FOXM1 on stemness *in vivo*. As shown in Table 1, both knocking down of AURKA and FOXM1 could reduce tumour initiation ability of breast cancer cells.

### AKI603 and thiostrepton synergistically inhibit the proliferation of BCSCs

**Figure 7:**
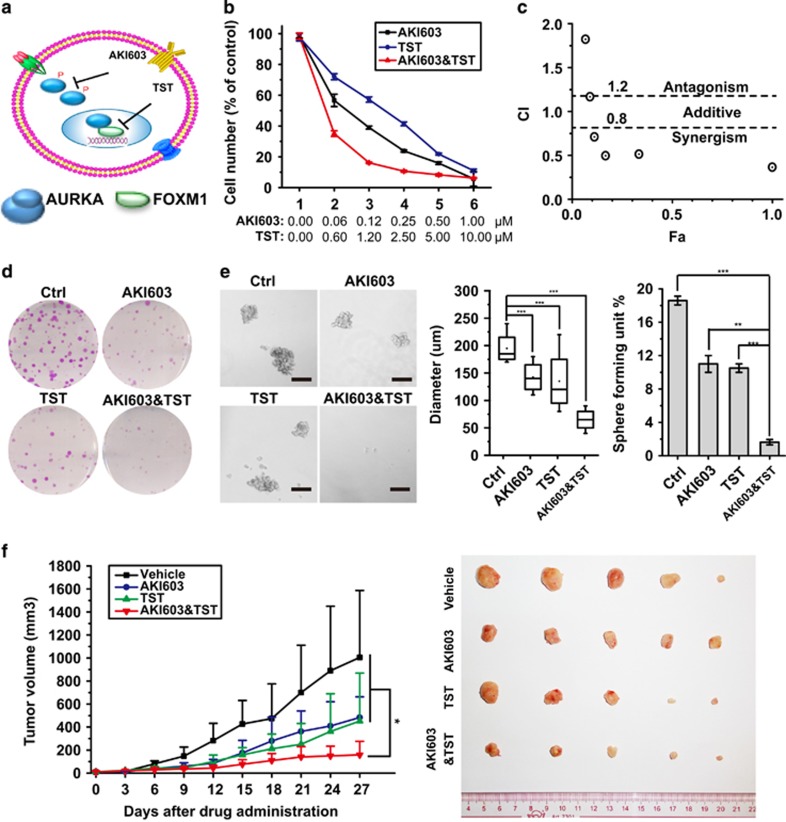
AKI603 and thiostrepton synergistically suppress BCSCs. (**a**) Scheme depicting the strategy to combine AURKA kinase inhibitor with FOXM1 inhibitor. (**b**) The cells were treated with various concentrations of drugs alone or in combination for 48 h. The DMSO-treated group proliferated normally. The growth inhibitory effects were determined by cell counting. The *Y* axis represents percent of viable cell number of drug-treated group versus DMSO-treated group. (**c**) CI value was calculated from data obtained in **b**. (**d**) MDA-MB-231 cells were treated 48 h with 0.12 μM AKI603, 1.2 μM thiostrepton alone, or in combination. Then, 500 cells were seeded into six-well plate and incubated with growth medium containing 0.12 μM AKI603, 1.2 μM thiostrepton alone, or in combination. After culture for 7 days, colonies were stained using crystal violet and the graphs were captured by invert microscope. (**e**) MDA-MB-231 cells were treated 48 h with 0.12 μM AKI603, 1.2 μM thiostrepton alone, or in combination. Then, 500 cells were seeded into low adhesion six-well plate and incubated with sphere medium containing 0.12 μM AKI603, 1.2 μM thiostrepton alone, or in combination. After culture for 7 days, spheres (diameter >50 μm) were analysed. (**f**) Nude mice bearing MDA-MB-231 xenograft tumours were treated with AKI603, thiostrepton, or the combinational therapy daily by intragastric administration for 14 days. The tumour volume was measured and calculated as shown. The above assays were repeated at least three times independently and results presented as mean±s.d. **P<0.01; ***P<0.001 significance by Student's *t*-test (two tailed).

Most of AURKA kinase inhibitors failed in the initial phases of clinical trials; however, very little is known about drug resistance mechanisms for these inhibitors. Recently, this drug failure has been proposed to be caused by the non-kinase dependent *trans*activating function of nuclear AURKA.^13^ Our present findings propose that nuclear AURKA drives a FOXM1 activation-mediated positive feedback loop that promotes the tumorigenicity and self-renewal of BCSCs and suggest that combined inhibition of AURKA and FOXM1 may effectively block the feedback loop and simultaneously inhibit the kinase and non-kinase function of AURKA (Figure 7a). To test this conjecture, we studied the ability of the novel AURKA inhibitor AKI603^8^ and the established FOXM1 inhibitor thiostrepton^30^ to function together in repressing the proliferation of MDA-MB-231 cell. To this end, proliferative analysis was performed to evaluate the potential synergistic interactions between AKI603 and thiostrepton in a fixed ratio (1:20). The results showed that the combinational treatment resulted in a more potent growth inhibition of MDA-MB-231 cells than that with the single drug alone (Figure 7b). These data were then subjected to combination index (CI) analysis.^31^ As shown in Figure 7c, the result revealed that AKI603 and thiostrepton acted synergistically to inhibit MDA-MB-231 cell proliferation.

We next investigated if the AURKA and FOXM1 inhibitors also act synergistically to suppress proliferation and self-renewal of BCSC. The combinational treatment exerted a greater suppression on colony formation compared with single agents alone (Figure 7d). Moreover, we gauged the effects of combining AKI603 and thiostrepton on mammosphere formation using MDA-MB-231 cells. As shown in Figure 7e, AKI603 alone (*P*<0.001) or thiostrepton alone (*P*<0.001) moderately reduced mammosphere number and size compared with the control, whereas the combinational treatment significantly reduced mammosphere number and size.

Finally, we evaluated the *in vivo* anticancer effects of AURKA and FOXM1 inhibitors in a xenograft model. Nude mice habouring MDA-MB-231 xenograft tumours were subjected to AKI603, thiostrepton, or the combination every day by intragastric administration for 14 days. As shown in Figure 7f, tumour volumes in the combinational treatment group were significantly smaller than those in the single drug groups and the control group. Taken together, these results indicated that AURKA inhibitor AKI603 and FOXM1 inhibitor thiostrepton synergistically inhibit the growth of BCSCs.

## Discussion

AURKA is one of the three highly conserved serine/threonine Aurora kinases (AURKA, -B and -C). It was originally identified as a mitotic kinase, which has essential roles in control of centrosome maturation/separation, bipolar spindle formation and G2/M progression to ensure the fidelity of mitosis.^4^ The role of AURKA in cancer is underscored by the fact that it is amplified and overexpressed in a multitude of human tumour types.^32^ In breast cancer, AURKA is amplified in about 12% of all primary tumours^32^ and its mRNA overexpressed in 62% of breast carcinomas.^33^ In addition, overexpression of AURKA is also observed in 94% of invasive ductal adenocarcinomas of the breast.^34^

Tumours are histologically heterogeneous, with different sub-populations of cancer cells exhibiting distinct molecular profiles and phenotypes.^35^ Although chemotherapy can eliminate most cells in a tumour, cancer stem cells survive and contribute to tumour drug resistance, recurrence and repopulation.^36^ This study supports a new role for AURKA in promoting cancer stem cell-like properties and anticancer chemotherapeutic drug resistance.^8, 13^ Accordingly, nuclear AURKA possesses stem cell-like characteristics, including enhanced mammosphere-forming ability, sub-population of CD44^lo^/CD24^hi^ breast cancer cells *in vitro* and self-renewal capacity in *in vivo* models.^8, 13^ In agreement, a recent study has also identified AURKA as a crucial transcriptional target of the pluripotency factor Nanog in embryonic and cancer stem cells.^37^

In here, we further uncover a positive feedback signalling loop between AURKA and FOXM1 essential for BCSC self-renewal and show that AURKA is recruited to the proximal *FOXM1* promoter by FOXM1 itself to function as a co-activator to enhance FOXM1 transcription. Interestingly, the kinase activity of AURKA is not involved in this nuclear function of AURKA. In concordance, we have previously shown that AURKA contains three putative nine amino-acid *trans*activation domains and is capable of promoting gene expression as a transcription factor through participating in assembling RNA polymerase II transcription machinery.^13^ A DNA-binding domain of AURKA has not been identified, and it is likely that AURKA does not bind to target genes directly and is recruited by transcription factors, such as FOXM1, to target genes to modulate transcription.^13^

Given the importance of AURKA in promoting tumorigenesis and cancer stem cell phenotypes, it is not surprising that about 30 Aurora kinase inhibitors are currently at different stages of pre-clinical and clinical development, and these AURKA inhibitors include MLN8237, CCT129202 and AT9283.^9, 12, 38, 39, 40^ However, a substantial number of AURKA inhibitors, such as MK-0457 (also called VX680, Vertex/Merck), AZD 1152 (Astra Zeneca), PHA 680632 (Nerviano), SU6668 (Sugen/Pfizer) and R763 (Merck/Serono), have failed, despite being considered successful in pre-clinical tests, suggesting that the mechanism of action for AURKA in cancer cells is more complex than our current understanding. Hitherto, the mechanism of resistance to AURKA inhibitors have not been identified, although some studies have advocated that mutations in the targeted kinase domain of Aurora kinase and the overexpression of drug resistance genes may be involved.^41^ Consistent with previous findings,^13^ we have showed here and also previously that the failure of some of these kinase inhibitors for AURKA may be caused by the non-kinase dependent *trans*activating function of AURKA in the nucleus that enhances the cancer stem cell properties. In agreement with this notion, AURKA nuclear staining has previously been reported in malignant tissues, and importantly, has been a predictor of poor clinical outcome.^42, 43, 44^

In the current work, we demonstrate that the FOXM1 inhibitor thiostrepton^30^ is particularly effective in combining with AURKA inhibitors to limit the tumorigenicity and self-renewal of BCSCs by synergistically suppressing the AURKA activity and disrupting the nuclear AURKA-FOXM1-positive feedback loop. This represents a promising therapeutic strategy for targeting both the kinase and non-kinase functions of AURKA to eliminate BCSCs. Besides that, our study also offers insights into the mechanism of action of nuclear AURKA as well as its downstream transcriptional network. Nevertheless, to devise better chemotherapeutic strategies to target AURKA to eliminate cancer stem cells, a comprehensive understanding of the nuclear AURKA molecular network and nucleus translocation mechanism is required.

In summary, our work has uncovered a previous unknown positive feedback loop between AURKA and FOXM1 that promotes BCSC phenotypes and drug resistance. We showed here that nuclear AURKA is recruited by FOXM1 to *trans*activate the expression of target genes, which also include FOXM1, whereas AURKA itself is also a downstream transcriptional target of FOXM1. This work further supports the novel non-canonical role of AURKA as a transcription factor as well as a promoter of the tumorigenicity and self-renewal of BCSCs. Crucially, we demonstrated that the AURKA inhibitor AKI603 and the FOXM1 inhibitor thiostrepton can effectively inhibit the tumorigenicity and self-renewal of BCSCs through synergistically inhibiting the AURKA activity and disrupting the positive feedback loop mediated by the non-kinase function of nuclear AURKA. These findings suggest a viable and novel therapeutic strategy to effectively inhibit the cytoplasmic and nuclear AURKA functions to eliminate BCSCs and to overcome AURKA inhibitor resistance.

## Materials and methods

### Cell lines and tissue culture conditions

Human breast cancer cell lines MDA-MB-231, SUM-149, MCF-7 and MCF-10 A were acquired from the American Type Culture Collection (Beijing Zhongyuan Ltd., Beijing, China), and cultured in media as recommended by the provider. Epirubicin-resistant MCF-7 (MCF-7-EpiR) cells have been described previously.^45, 46, 47^ MCF-7-EpiR cells were cultured in Dulbecco’s modified Eagle’s medium (Gibco, ThermoFisher Scientific, Shatin, Hong Kong) supplemented with 10% foetal bovine serum (Hyclone, ThermoFisher Scientific, Beijing, China). All of these cell lines were authenticated by the standard short tandem repeat DNA typing.

#### Tissue samples

All clinical specimens were derived from breast cancer patients from Sun Yat-Sen University Cancer Center, Guangzhou, China, with patients’ consent and approval from the Sun Yat-Sen University Cancer Center Institute Research Ethics Committee.

#### Plasmids and transfection

Complementary DNA encoding human AURKA (WT) and human FOXM1 were generated by PCR amplification and subcloned into the pBabe-puro vector (Invitrogen, Beijing, China) for expression studies. AURKA mutant constructs were generated using the Quick Change Site directed mutagenesis kit (Stratagene, ThermoFisher Scientific) according to the manufacturer’s protocols. Plasmids were transfected into cells using Lipofectamine 2000 (Invitrogen) following the manufacturer’s instructions. The primers used are shown in Supplementary Table S2.

#### Gene knockdown using shRNA

Gene silencing was performed using specific shRNAs delivered by a lentiviral system acquired from Sigma-Aldrich (Shanghai, China), following the instructions provided. Briefly, to yield lentiviruses containing specific shRNA sequences, 293T cells were co-transfected with 2.5 μg pMD2.G and 7.5 μg psPAX2 packaging plasmids and 10 μg of the pLKO.1 plasmid containing the specific shRNA for 24 h. The lentivirus containing cultured medium was collected and stored at −80°C as aliquots until further use. To deliver the specific shRNA construct, approximately 10% confluent cells were incubated with the lentivirus bearing specific shRNA in growth medium containing 8 mg/ml polybrene at 37° for 24 h. The transduced cells were then selected with 2 mg/ml puromycin. All shRNA constructs used are shown in Supplementary Table S2.

#### Real-time RT–PCR

Total RNA was extracted using TRIzol reagent (Invitrogen), and used for generating cDNA using SuperScript III RT (Invitrogen) in the presence of oligo-dT primers. Real-time reverse transcriptase–PCR was performed using Platinum SYBRGreen qPCR SuperMix (Invitrogen), with glyceraldehyde 3-phosphate dehydrogenase as the internal control. The primers used are listed in Table 1.

#### CD24 and CD44 expression analysis

Cells were harvested and incubated with anti-human CD24 phycoerythrin (eBioscience, Gene Company Ltd, Shanghai, China) and anti-human CD44-FITC (eBioscience, Gene Company Ltd) at 4 °C. The cells were then resuspended in 1 ml of phosphate-buffered saline and analysed using a FACScan cytometer (Beckon Dickinson, Oxford, UK).

#### Immunofluorescence staining

Cells were fixed in 2% paraformaldehyde at room temperature for 20 min and permeabilised in 0.5% Triton X-100 in phosphate-buffered saline for 10 min. Slides were then incubated with the primary antibody for 60 min. The antibodies used were anti-AURKA antibody (ab13824, Abcam, Hangzhou, China, 1:200 dilution), anti-FOXM1 antibody (sc502, Santa Cruz Biotechnology, Shanghai, China, 1:500 dilution). The secondary antibodies were conjugated to either Alexa-488 or Alexa-546 (Molecular Probes, Invitrogen, 1:200 dilution). Nuclei were stained with DAPI (Sigma-Aldrich) and visualised with a Leica TCS SP5 confocal microscope (Leica Microsystems, Mannhein, Germany) equipped with a 63x oil immersion objective and the LAS-AF software (Santa Cruz Biotechnology).

#### Western blot analysis

Cells were lysed in RIPA (radioimmunoprecipitation assay) buffer and the protein concentrations determined by the Bradford assay. Equal amounts of cell extracts were subjects to electrophoresis on sodium dodecyl sulphate–polyacrylamide gel and blotted onto nitrocellulose membrane (Millipore, Merck, Shanghai, China). After protein transfer, the membranes were blocked and then incubated with glyceraldehyde 3-phosphate dehydrogenase (Ambion, ThermoFisher Scientific), β-actin (Santa Cruz Biotechnology, Gene Company Ltd), p-AURKA (Thr288; Cell Signaling Technology, Gene Company Ltd), AURKA (Upstate, Gene Company Ltd), HA-tag (Sigma), c-Myc (Santa Cruz Biotechnology), SOX2 (Epitomics, Abcam, Hang Zhou, China) and Nanog (Epitomics, Abcam) primary antibodies at 4 ° overnight. The membranes were then incubated for 1 h at room temperature with the appropriate secondary antibodies. Proteins were detected with an enhanced chemiluminescence kit (Pierce, ThermoFisher Scientific).

#### Mammosphere formation assay

Mammosphere formation analyses were performed as described previously.^13^ See Supplementary Materials and Methods for details.

#### Luciferase reporter assay

Cells plated at a density of 1 × 10^5^per well in 24-well plates overnight were transfected with a FOXM1 or AURKA promoter-driven luciferase construct or the control luciferase construct using Lipofectamine 2000. Twenty-four hours after transfection, cells were collected and Firefly and Renilla luciferase activities measured using a dual luciferase kit (Promega, Beijing, China). Firefly luciferase data for each sample were normalised against Renilla activity.

#### Chromatin immunoprecipitation (ChIP)

ChIP assays were carried out according to the manufacturer’s protocol (EZ-Magna ChIP kit, Millipore, Merck). AURKA and FOXM1 antibodies mentioned were used for ChIP analysis. For sequential ChIP (Re-ChIP), the first elute immunoprecipitated by anti-AURKA was further immunoprecipitated with either anti-FOXM1 or IgG antibodies. The percentage of chromatin-bound recovered DNA was quantified against DNA input. Primers used for the amplification of the precipitated DNA are listed in Supplementary Table S2.

#### CI calculation

Data acquired from the cell viability assay were used to calculate the CI. The CI was analysed with the CompuSyn software (CompuSyn, Paramus, NJ, USA) using the average fraction of cells that responded to each drug. CI values of <0.8, between 0.8 and 1.2, and >1.2 were defined as synergistic, additive and antagonistic, respectively.^48^

#### Immunohistochemical staining and statistical analysis

Following informed consent and in accordance with the guidelines of the Institutional Review Boards, breast cancer specimens were collected, with informed consent and in accordance with the guidelines of the Institutional Review Boards, from patients undergoing surgery at the Third Affiliated Hospital of Sun Yat-Sen University, China. Paraffin-embedded tissue blocks were sectioned for immunohistochemistry as described previously.^13^ The deparaffinised sections were incubated in H_2_O_2_ (3%) for 10 min, blocked in 1% bovine serum albumin for 60 min and incubated with an anti-AURKA antibody (1:400 dilution) or an anti-FOXM1 antibody (1:100 dilution) at 4 °C overnight. See Supplementary Materials and methods for details.

#### Tumour growth in xenografts

Animal work was performed with approval from the local Institutional Animal Care and Use Committee (Sun Yat-Sen University Cancer Center). MDA-MB-231 cells were treated with 200 ng/ml Dox for 7 days, and 5 × 10^5^ cells injected into the right flank of 4-week-old female nude mice (*n*=9) as described previously.^13^ See Supplementary Materials and Methods for details.

#### *In vivo* tumour initiation assay

To evaluate effect of AURKA and FOXM1 on stemness, limiting dilution assays were performed in NOD/SCID mice. Cells were treated with 200 ng/ml Dox for 1 week, and injected subcutaneously into NOD/SCID mice at concentrations of 5 × 10^5^, 1 × 10^5^, 1 × 10^4^ and 1 × 10^3^cells per site. Eight mice were used in each experimental group. Tumour formation was checked every 4 days and the observation time was 8 weeks in total.

### Statistical analysis

Statistical analyses were performed using the SPSS software, version 16.0 (SPSS Inc., Chicago, IL, USA) and with GraphPad Prism 5.0 (GraphPad Software, Inc., LaJolla, CA, USA). Kruskal–Wallis test, followed by a Dunn multiple comparison test, was used to compare mammosphere size distributions. The unpaired Student's *t*-test was used to perform statistical analysis between two groups. The analysis of variance test and the least significant difference test were used for conducting multiple comparisons. The level of significance was set at *P*<0.05

## Figures and Tables

**Table 1:** Limiting dilution assays to evaluate effect of AURKA and FOXM1 on stemness

*Group*	*Tumour incidence*			
	*5 × 10*^*5*^ *Cells*	*1 × 10*^*5*^*Cells*	*1 × 10*^*4*^ *Cells*	*1 × 10*^*3*^ *Cells*
shCtrl+oeCtrl	7/7	7/7	6/7	3/7
shAURKA+oeCtrl	8/8	6/8	3/8	1/8
shFOXM1+oeCtrl	7/7	6/7	3/7	0/7
shAURKA+oeFOXM1	8/8	7/8	5/8	3/8
shFOXM1+oeAURKA	8/8	6/8	4/8	1/8

Abbreviations: AURKA, Aurora kinase A; Dox, doxycycline; FOXM1, Forkhead box subclass M1; oe, overexpress target genes; sh, shRNA to knockdown target genes.

Cells were treated with 200 ng/ml Dox for 1 week, and were transplanted subcutaneously into NOD/SCID mice at concentrations of 5 × 10^5^, 1 × 10^5^, 1 × 10^4^ and 1 × 10^3^ cells per site.
