# Supplementary material for: FOXM1 recruits nuclear Aurora kinase A to participate in a positive feedback loop essential for the self-renewal of breast cancer stem cells
Source: Oncogene. 2017 Jan 23;36(24):3428–40. doi: 10.1038/onc.2016.490 (PMC5485180; doi:10.1038/onc.2016.490)
Supplement: Supplementary Information [file onc2016490x1.docx]

**Supplementary Materials and Methods**

**Immunohistochemical staining and statistical analyses**

Paraffin-embedded tissue blocks were sectioned for immunohistochemical staining. Paraffinembedded tissue specimens were sectioned, deparaffinised in xylene and rehydrated. Antigenic retrieval was processed with sodium citrate. The sections were then incubated in H_2_O_2_ (3%) for 10 min, blocked in 1% bovine BSA for 60 min and incubated with an anti-AURKA antibody (1:400 dilution) and anti-FOXM1 antibody (1:100 dilution) at 4℃ overnight. After incubation with the secondary antibody for 60 min, specimens were incubated with H_2_O_2_-diaminobenzidine until the desired stain intensity was developed. Sections were then counterstained with haematoxylin, dehydrated and mounted. Staining intensities and extents of AURKA and FOXM1 expression were graded as follows: negative (score 0), bordering (score 1), weak (score 2), moderate (score 3) and strong (score 4). Extent of staining was also grouped into quintiles according to the percentage of high-staining cells in the field: negative (score 0), ≤25% (score 1), 26–50% (score 2), 51–75% (score 3) and 76–100% (score 4). All immunohistochemical stainings were evaluated and scored by at least two independent pathologists.

**Mammosphere formation assay**

Single-cell suspension was obtained after trypsinisation. Clumped cells were excluded with a 40-mm sieve, and the suspension was analysed microscopically for single cellularity. The percentage of clumped cells was <5%. Single cells were plated in ultralow attachment 6-well plates at a low density of 1,000 viable cells/mL. Cells were maintained in DMEM/F12 (Gibco) supplemented with B27 (Invitrogen), 20 ng/mL EGF (Sigma-Aldrich), 20 ng/mL asic fibroblast growth factor (bFGF; BD Biosciences), and 4 mg/mL heparin (Sigma) for 7 days. The mammospheres were photographed using inverted microscope (Olympus). The iameters of the mammospheres were calculated with the Image pro plus 6.0 software (Media ybernetics).

**Tumour growth in xenografts**

MDA-MB-231 cells (1×10^5^) were injected into the right flank of 4-week-old female nude mice (n = 5). On day 30, the mice (tumour size, ~50mm^3^) were randomly distributed into two groups that were treated intragastrically every day with either a vehicle control or with 50 mg/kg AKI603 dissolved in PEG300. The tumour volumes were measured by calipers. Other indicators of general health, such as body weight, feeding behavior, and motor activity, of each animal were also monitored. After administering the drug or vehicle for 14 days, the mice were fed for another 14 days. At day 59, the mice were sacrificed and the tumour xenografts were immediately dissected, weighed, stored, and fixed. All animal procedures were approved by the Institutional Animal Care and Use Committee of the Sun Yat-sen University Cancer Center.
