# Supplementary material for: FOXM1 recruits nuclear Aurora kinase A to participate in a positive feedback loop essential for the self-renewal of breast cancer stem cells
Source: Oncogene. 2017 Jan 23;36(24):3428–40. doi: 10.1038/onc.2016.490 (PMC5485180; doi:10.1038/onc.2016.490)
Supplement: Supplementary Information [file onc2016490x2.pdf]

Supplementary Table S1.

FOXM1

**Variables**	**High expression**	**Low expression**	**Total**	**P Value**
Age				
<60 years	89	73	162	0.56923
⩾60 years	55	52	107	
Lymph node metastasis				
Negative	45	64	109	0.00088
Positive	99	61	160	
Tumour grade				
1	38	31	69	0.40023
2	55	40	95	
3	51	54	105	

Aurora kinase A (AURKA)

**Variables**	**High expression**	**Low expression**	**Total**	**P Value**
Age				
<60 years	110	52	162	0.70413
⩾60 years	75	32	107	
Lymph node metastasis				
Negative	65	44	109	0.00758
Positive	120	40	160	
Tumour grade				
1	45	24	69	0.75211
2	67	28	95	
3	73	32	105	
