# Supplementary material for: FOXM1 recruits nuclear Aurora kinase A to participate in a positive feedback loop essential for the self-renewal of breast cancer stem cells
Source: Oncogene. 2017 Jan 23;36(24):3428–40. doi: 10.1038/onc.2016.490 (PMC5485180; doi:10.1038/onc.2016.490)
Supplement: Supplementary Table 1 [file onc2016490x3.pdf]

Table S2. Primers used in this study.

Name	Sense primer (5' to 3')	Anti-sense primer (5' to 3')	Application
AURKA mRNA RTq-PCR	TGGAATATGCACCACTTGGA	ACTGACCACCCAAAATCTGC	Real-Time PCR for AURKA mRNA
AURKA-promoter (-1507/+126)	ATGGTACCACCAGGGAAGATTAACTCG	ATACGCGTACACTCAGGGACGGAGGAA	AURKA promoter luciferase repertor construction
AURKA-promoter (-2047/+126)	ATGGTACCAAGTGCTGGGATTACGGG	ATACGCGTACACTCAGGGACGGAGGAA	AURKA promoter luciferase repertor construction
AURKA-promoter (-28/+126)	ATGGTACCAAGGCGTCGGGTTTGTTG	ATACGCGTACACTCAGGGACGGAGGAA	AURKA promoter luciferase repertor construction
AURKA-promoter (-968/+126)	ATGGTACCCTGGAAGGATGGAGGGAA	ATACGCGTACACTCAGGGACGGAGGAA	AURKA promoter luciferase repertor construction
AURKA-promoter FKHR mutant	CACTCGCCAGGTAGGGAGAAGCCTAA	TTAGGCTTCTCCCTACCTGGCGAGTG	FOXM1 binding motif mutant of AURKA promoter
ChIP-1 (-47/+98) FOXM1	GATTGGCGACGTTCCGTCA	TGGGAACCCCGGGGGATCC	ChIP/ReChIP Real-Time PCR (AURKA binds to FOXM1 promoter)
ChIP-2 (-769/-763) AURKA	GTGGCCCACCCCTAACTTCT	GTCCTCGTGTGCTCACCTGC	ChIP Real-Time PCR (FOXM1 binds to AURKA promoter)
ChIP-ctrl-1 (-2756/-2651) FOXM1	CCTATGCGTATCAACATC	AGAGTGAGAACAGCTCCA	ChIP/ReChIP Real-Time PCR (AURKA binds to FOXM1 promoter)
ChIP-ctrl-2 (-2047/-1912) (AURKA)	AAAGTGCTGGGATTACGGGC	TTAGCATGACGGTATCTGGCATG	ChIP Real-Time PCR (FOXM1 binds to AURKA promoter)
FOXM1 cDNA	ATGGATCCGCCACCATGAAAACTAGCCC	ATTCTAGACTACTTATCGTCGTCATCCT	FOXM1 overexpression plasmid construction
FOXM1 mRNA RTq-PCR	ATACGTGGATTGAGGACCACT	TCCAATGTCAAGTAGCGGTTG	Real-Time PCR for FOXM1 mRNA
FOXM1-promoter (+18/+1034)	ACTGAAAGCTCCGGTGCCA	CGACGCGTCGTTCACGGAACTTTGGAA	FOXM1 promoter luciferase repertor construction
FOXM1-promoter (-1121/+1034)	CCGCTCGAGCGCCCTTTCCTGTCCTACC	CGACGCGTCGTTCACGGAACTTTGGAA	FOXM1 promoter luciferase repertor construction
FOXM1-promoter (-47/+1034)	CCGCTCGAGCCGGATTGGCGACGTTCCC	CGACGCGTCGTTCACGGAACTTTGGAA	FOXM1 promoter luciferase repertor construction
FOXM1-promoter FKHR mutant	GCCAATTTCAGGGAGCGGAACAA	TTGTTCCGCTCCCTGAAAATTGGC	FOXM1 binding motif mutant of FOXM1 promoter
GAPDH mRNA RTq-PCR	ATGGGGAAGGTGAAGGTCGG	TGGAGGCCATGTGGGCCATG	Real-Time PCR control
shAURKA-1	CCGGGGCAACCAGTGTACCTCATCTCGA	ATTAAAAAGGCAACCAGTGTACCTCATC	shAURKA-1 plasmid construction
shAURKA-2	CCGGATTCTTCCCAGCGCGTTCCCTCGA	AATTAAAAAATTCTTCCCAGCGCGTTCC	shAURKA-2 plasmid construction
shFOXM1-1	CCGGGCCCAACAGGAGTCTAATCAACTC	AATTAAAAAGCCCAACAGGAGTCTAATC	shFOXM1-1 plasmid construction
shFOXM1-2	CCGGGCCAATCGTTCTCTGACAGAACTC	AATTAAAAAGCCAATCGTTCTCTGACAG	shFOXM1-2 plasmid construction
siAURKA-1	AUGCCCUGUCUUACUGUCA		Transient knock down AURKA
siAURKA-2	GGCAACCAGTGTACCTCAT		Transient knock down AURKA
siCtrl	UUCUCCGAACGUGUCACGU		Transient knock down gene control
siFOXM1-1	GCACTATCAACAATAGCCTAT		Transient knock down FOXM1
siFOXM1-2	GCCAATCGTTCTCTGACAGAA		Transient knock down FOXM1
