# Supplementary material for: FOXM1 recruits nuclear Aurora kinase A to participate in a positive feedback loop essential for the self-renewal of breast cancer stem cells
Source: Oncogene. 2017 Jan 23;36(24):3428–40. doi: 10.1038/onc.2016.490 (PMC5485180; doi:10.1038/onc.2016.490)
Supplement: Supplementary Table 2 [file onc2016490x4.pdf]

Figure S1

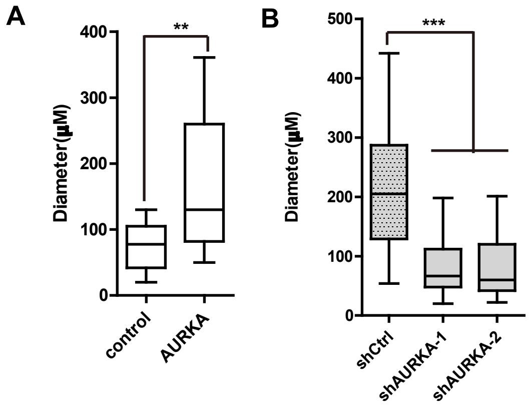

Figure S1. AURKA regulates the size of individual tumorspheres. (A) AURKA overexpression increase the size of individual tumorspheres compared to control in MCF-7 cells. (B) AURKA knockdown decrease the size of individual tumorspheres compared to control in MDA-MB-231 cells.
