# Supplementary material for: FOXM1 recruits nuclear Aurora kinase A to participate in a positive feedback loop essential for the self-renewal of breast cancer stem cells
Source: Oncogene. 2017 Jan 23;36(24):3428–40. doi: 10.1038/onc.2016.490 (PMC5485180; doi:10.1038/onc.2016.490)
Supplement: Supplementary Figure 2 [file onc2016490x6.pdf]

Figure S3

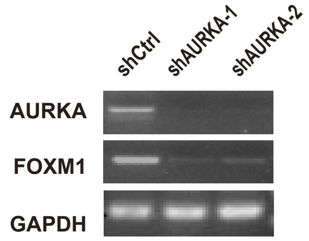

Figure S3. Semi-quantitative RT PCR detects mRNA levels of AURKA and FOXM1 in control (shCtrl) and AURKA (shAURKA) knockdown MDA-MB-231 cells.
