# Supplementary material for: FOXM1 recruits nuclear Aurora kinase A to participate in a positive feedback loop essential for the self-renewal of breast cancer stem cells
Source: Oncogene. 2017 Jan 23;36(24):3428–40. doi: 10.1038/onc.2016.490 (PMC5485180; doi:10.1038/onc.2016.490)
Supplement: Supplementary Figure 3 [file onc2016490x7.pdf]

Figure S4

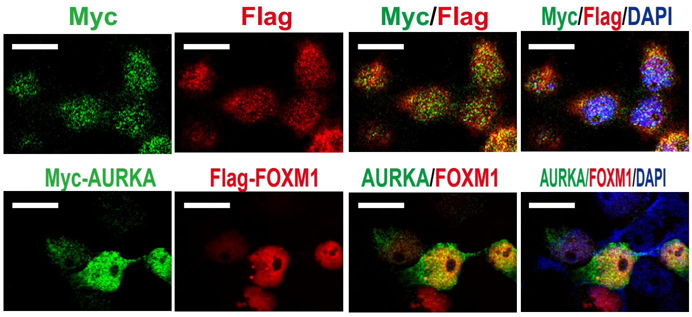

Figure S4. Nuclear AURKA directly binds to FOXM1 to trans-activate FOXM1 expression. Immunofluorescence assay to detect intracellular localization of AURKA and FOXM1. Scale bar, 20 μm.
