# Supplementary material for: FOXM1 recruits nuclear Aurora kinase A to participate in a positive feedback loop essential for the self-renewal of breast cancer stem cells
Source: Oncogene. 2017 Jan 23;36(24):3428–40. doi: 10.1038/onc.2016.490 (PMC5485180; doi:10.1038/onc.2016.490)
Supplement: Supplementary Figure 4 [file onc2016490x8.pdf]

Figure S5

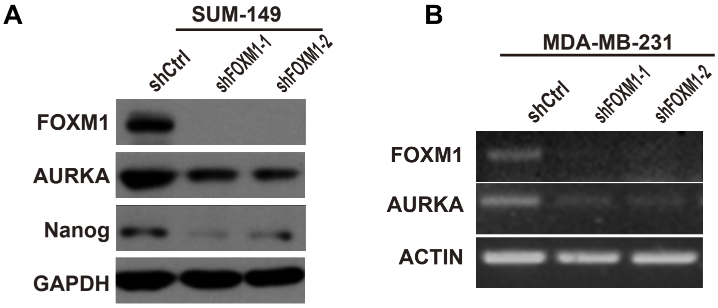

Figure S5. FOXM1 directly activates AURKA expression at transcriptional level. (A) Western blot analysis with indicated antibodies in control (shCtrl) and FOXM1 (shFOXM1) knockdown SUM-149 cells. (B) Semi-quantitative RT PCR detects mRNA levels of FOXM1 and AURKA in control (shCtrl) and FOXM1 (shFOXM1) knockdown MDA-MB-231 cells.
