# Supplementary material for: FOXM1 recruits nuclear Aurora kinase A to participate in a positive feedback loop essential for the self-renewal of breast cancer stem cells
Source: Oncogene. 2017 Jan 23;36(24):3428–40. doi: 10.1038/onc.2016.490 (PMC5485180; doi:10.1038/onc.2016.490)
Supplement: Supplementary Figure 5 [file onc2016490x9.docx]

**Supplemental Figure Legends**

Figure S1. AURKA regulates the size of individual tumourspheres. (A) AURKA overexpression increase the size of individual tumourspheres compared to control in MCF-7 cells. (B) AURKA knockdown decrease the size of individual tumorspheres compared to control in MDA-MB-231 cells.

Figure S2. FOXM1 knockdown decreases the size of individual tumourspheres compared to control in MDA-MB-231 cells.

Figure S3. Semi-quantitative RT PCR detects mRNA levels of AURKA and FOXM1 in control (shCtrl) and AURKA (shAURKA) knockdown MDA-MB-231 cells.

Figure S4. Nuclear AURKA directly binds to FOXM1 to trans-activate FOXM1 expression. Immunofluorescence assay to detect intracellular localization of AURKA and FOXM1. Scale bar, 20 μm.

Figure S5. FOXM1 directly activates AURKA expression at transcriptional level. (A) Western blot analysis with indicated antibodies in control (shCtrl) and FOXM1 (shFOXM1) knockdown SUM-149 cells. (B) Semi-quantitative RT PCR detects mRNA levels of FOXM1 and AURKA in control (shCtrl) and FOXM1 (shFOXM1) knockdown MDA-MB-231 cells.
